# PRDM1-mediated epigenetic and transcriptional repression mechanisms: a key hub in immune differentiation, tumor progression, and inflammatory responses

**DOI:** 10.3389/fimmu.2026.1856181

**Published:** 2026-06-18

**Authors:** Zongliang Xu, Shiyuan Liu, Yizhuo Fu, Yuqin Zhang, Wenzhi Shen, Huan Liu

**Affiliations:** 1 School of Clinical Medicine (Affiliated Hospital), Jining Medical University, Jining, Shandong, China; 2 Shandong Provincial Precision Medicine Laboratory for Chronic Non-communicable Diseases, Institute of Precision Medicine, Jining Medical University, Jining, Shandong, China; 3 Blood Transfusion Department, Affiliated Hospital of Jining Medical University, Jining Medical University, Jining, Shandong, China

**Keywords:** immune differentiation, inflammatory response, PRDM1, transcriptional repression, tumor progression

## Abstract

PRDM1 is a key transcriptional repressor that plays a vital role in B- and T-cell differentiation, immune regulation, and tumor suppression. PRDM1 functions through its C2H2 zinc finger domains and proline-rich region, which mediate transcriptional repression by recruiting co-repressors like G9a and HDACs. Its expression is regulated by multiple signaling pathways, including JAK/STAT3 and CD40L, which control immune cell differentiation and function. PRDM1 is involved in cancer progression by regulating tumor suppression and metastasis, as seen in hepatocellular, colorectal, and breast cancers. Additionally, PRDM1 is implicated in autoimmune diseases such as lupus and rheumatoid arthritis, where it modulates B-cell differentiation and immune responses. Emerging evidence suggests that single nucleotide polymorphisms in the PRDM1 could serve as valuable biomarkers for disease diagnosis and prognosis. This review highlights the multifaceted roles of PRDM1 in disease pathogenesis and explores its potential as a therapeutic target for cancer and immune-related diseases.

## 1 Introduction

PR domain zinc finger protein 1 (PRDM1), initially discovered in 1991 as a virus-induced, specific transcriptional repressor that negatively regulates the transcription of the β-interferon gene, was named PRDI-binding factor I (PRDI-BF1) (1). In 1994, Turner and Davis discovered a new gene significantly induced during B cell differentiation into plasma cells, named B lymphocyte-induced maturation protein-1 (Blimp-1). Blimp-1 inhibits the transcription of proliferation genes such as c-Myc in B cells, induces immunoglobulin secretion, and expresses plasma cell markers, thus promoting B cell differentiation (2). Subsequent sequence analysis showed that Blimp-1 and PRDI-BF1 encoded the same protein, leading to the renaming of the gene as PRDM1 (3).

At the molecular level, PRDM1 directly binds to DNA through its zinc finger domains and recruits multiple epigenetic regulators to form transcriptional repression complexes, thereby silencing target gene expression and functioning as a key regulator of cell fate determination. PRDM1 has attracted considerable attention for its roles in inflammation and autoimmune diseases. By regulating the differentiation of B cells into plasma cells and modulating T-cell effector functions, PRDM1 is essential for maintaining immune homeostasis and preventing excessive immune activation. Dysregulation of *PRDM1* expression or function has been closely associated with multiple immune-related diseases, including systemic lupus erythematosus, rheumatoid arthritis, and inflammatory bowel disease, further underscoring its central role in immune regulation (4). Beyond its well-established role in the immune system, PRDM1 also plays critical roles in tumor initiation and progression. It acts as a tumor suppressor in certain cancer types; however, in other contexts, PRDM1 can promote tumor progression, immune evasion, and metastasis, highlight its context-dependent functions and establish it as a complex and pivotal regulatory node in tumor biology (5). Although substantial progress has been made in understanding PRDM1, its functional heterogeneity and underlying molecular mechanisms across different cell types and tissue microenvironments remain incompletely understood. Particularly in the context of cancer, how PRDM1 mediates epigenetic regulation to switch between tumor-suppressive and tumor-promoting roles remains a critical unresolved question. Based on this, this review systematically summarizes the structural features of PRDM1 and its transcriptional and epigenetic regulatory mechanisms, with a particular focus on its roles in immune regulation, tumor progression, and inflammation-related diseases. This work aims to provide a theoretical basis for mechanistic studies and the development of precision therapeutic strategies targeting PRDM1.

## 2 Structure and function

*PRDM1* is located on the long arm of chromosome 6 (6q21–q22.1), an area whose gene deletions are closely associated with the occurrence of various malignancies, especially B-cell non-Hodgkin lymphoma, potentially serving as a region rich in tumor suppressor genes (6). *PRDM1* belongs to the PRDM family, which includes 17 members from *PRDM1* to *PRDM17*. Recently, it was discovered that *ZFPM1* and *ZFPM2* also belong to this family (7–9). *PRDM9* represents a mechanistically distinct PRDM-family model. Unlike *PRDM1*, PRDM9 contains an enzymatically active PR/SET domain and a rapidly evolving zinc-finger array that recognizes recombination hotspot motifs. After DNA binding, PRDM9 facilitate programmed double-strand break formation and meiotic recombination. Ladias et al. further linked cancer-associated *PRDM9* motif architecture with breakpoint regions and genomic instability, suggesting that PRDM9 may influence cancer biology through recombination-associated DNA damage rather than PRDM1-like transcriptional repression (10). PRDM1 primarily contains several structural domains; (1) Five C2H2 zinc finger domains, (2)A PR domain (36–166), (3)A proline-rich region.

The zinc finger domains of PRDM1 mediate DNA binding. Zinc ions interact with the protein through cysteine and histidine residues, forming a stable zinc finger structure. Further research has shown that the first two zinc fingers of PRDM1 are sufficient to recognize the PRDI element for sequence-specific binding to DNA (11). As a direct DNA-binding protein, the PR domain of PRDM1 shares high homology with the SET domain in methyltransferases but lacks methyltransferase activity. Mechanistically, PRDM1 exerts transcriptional repression through recruitment of the histone methyltransferase G9a (EHMT2). Using co-immunoprecipitation and chromatin immunoprecipitation (ChIP) assays, Györy et al. demonstrated that PRDM1 physically associates with G9a and facilitates its recruitment to PRDM1-bound target promoters. G9a subsequently catalyzes the deposition of histone H3 lysine 9 dimethylation (H3K9me2), a hallmark of transcriptionally repressive chromatin. Enrichment of H3K9me2 promotes the binding of heterochromatin protein 1 (HP1), leading to local chromatin condensation, reduced accessibility of transcription factors and RNA polymerase II, and ultimately stable silencing of target genes (12). Notably, disruption of G9a methyltransferase activity by expression of a catalytically inactive mutant markedly attenuated PRDM1-mediated transcriptional repression, demonstrating that G9a-dependent H3K9 methylation is functionally required for PRDM1-driven epigenetic silencing (13).

Furthermore, the proline-rich region at the N-terminus can recruit histone deacetylases (HDACs) and core repressors from the Groucho family to form transcriptional repression complexes, thus enabling transcriptional silencing (14, 15). *PRDM1* also mediates transcriptional repression through HDAC-dependent chromatin remodeling. Yu et al. demonstrated that PRDM1 represses the *MYC* promoter by recruiting HDAC activity. Treatment with the HDAC inhibitor trichostatin A partially relieved this repression, and ChIP analysis showed reduced histone H3 acetylation at the MYC promoter after PRDM1 expression, supporting a functional role for histone deacetylation in PRDM1-dependent gene silencing (15). Regarding HDAC isoform specificity, available evidence supports the involvement of class I HDAC-associated repression machinery. HDAC2 has been directly implicated in *PRDM1*-mediated repression in CD8^+^ T cells, whereas HDAC1/HDAC2-associated complexes have been reported in PRDM1-related repression systems (16). By contrast, direct evidence for HDAC3 as a *PRDM1*-recruited HDAC isoform remains limited.

PRDM1-mediated repression may also involve chromatin-remodeling co-repressor complexes, including NuRD. NuRD is mechanistically important because it combines HDAC1/2-mediated histone deacetylation with CHD3/CHD4-dependent ATP-driven nucleosome remodeling, thereby linking histone modification to chromatin accessibility control (17). Minnich et al. showed that *PRDM1* associates with multiple chromatin regulatory complexes, suggesting that *PRDM1*-dependent repression extends beyond simple DNA binding to coordinated chromatin remodeling and stable gene silencing (18). However, direct evidence for PRDM1–NuRD recruitment at cancer-specific target genes remains limited. Direct evidence that PRDM1 controls enhancer-promoter looping or higher-order chromatin architecture remains limited. Most current data support PRDM1-dependent regulation of chromatin accessibility, histone modifications, and recruitment of chromatin-modifying complexes.

PRDM1 has also been demonstrated to function as a direct transcriptional activator in a context-dependent manner during embryonic lineage specification, particularly in neural and sensory progenitor development. Prajapati et al. showed that during early chick embryogenesis, *PRDM1* is transiently expressed in the epiblast and subsequently becomes enriched in emerging neural and sensory progenitor populations. Mechanistically, PRDM1 directly binds to the promoter regions of key lineage-specifying genes, which are essential regulators of neural plate and sensory progenitor identity. Notably, these promoters are initially maintained in a transcriptionally repressed chromatin state, characterized by the presence of H3K9me3, a well-established repressive histone mark. PRDM1 activation does not occur through indirect depression, but rather through a direct chromatin-remodeling mechanism. Specifically, PRDM1 recruits the histone demethylase KD4MA to target promoters, resulting in the removal of H3K9me3 and the establishment of a more permissive chromatin environment for transcriptional activation. These findings provide strong experimental evidence that PRDM1 is not exclusively a transcriptional repressor, but can also act as a direct epigenetic activator by binding target promoters and remodeling local chromatin states in a developmental context (19).

### 2.1 PRDM1α and PRDM1β, isoform-specific structure

**Figure 1:**
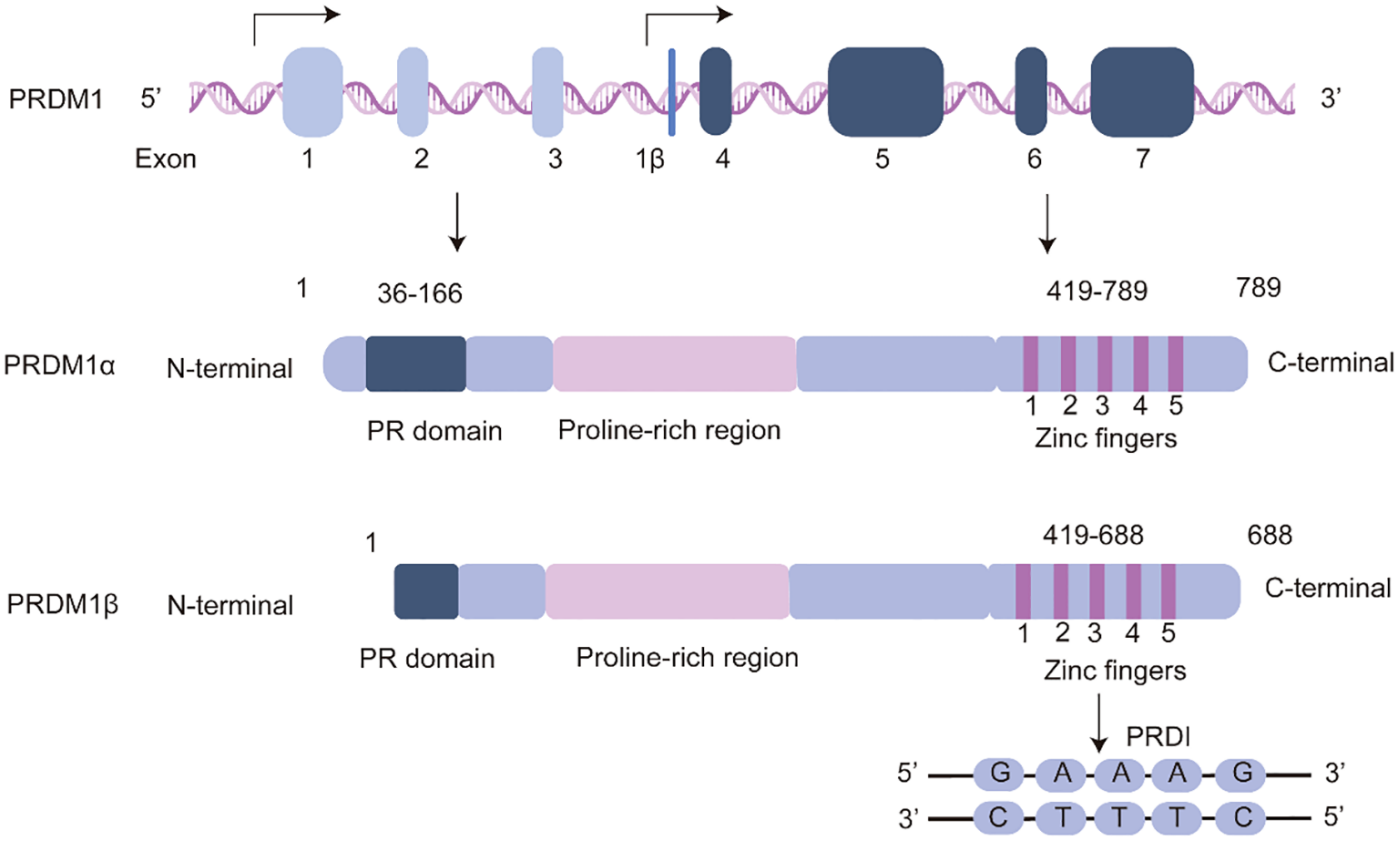
Structural domains and isoforms of PRDM1. PRDM1 consists of multiple exons and alternative transcription start sites that generate two major isoforms, PRDM1α and PRDM1β. PRDM1α is the full-length isoform containing an N-terminal PR domain, a proline-rich region, and five C2H2 zinc-finger domains at the C-terminus. PRDM1β is transcribed from an alternative promoter and lacks the first 101 amino acids of the N-terminus, resulting in partial loss of the PR domain but retention of the DNA-binding zinc-finger region. The C-terminal zinc fingers, particularly ZF1-ZF2, mediate sequence-specific binding to PRDI-like motifs (GAAAG-rich sequences), enabling transcriptional regulation of genes involved in immune regulation, differentiation, and tumor biology.

Györy et al. identified a functionally impaired PRDM1 transcription repressor in myeloma cell lines. Alternative transcription initiation of PRDM1 results in two functionally distinct protein isoforms. PRDM1α (full-length protein) is the initial gene product, while PRDM1β, transcribed from an alternative promoter between exons 1 and 4, lacks the first 101 amino acids, which include the complete PR domain. Structurally similar to PRDM2 (RIZ), PRDM1β has reduced transcriptional activity and only 20% of the transcriptional repressive capacity of PRDM1α, leading to abnormal transcriptional regulation in myeloma (**Figure 1**). This may be related to the development of myeloma (20). PRDM1β may still recognize some target loci through its zinc-finger domains, but its disrupted PR domain limits the ability to establish a complete and stable repressive chromatin state. Thus, relative enrichment of PRDM1β or loss of PRDM1α may weaken repression of MYC, PAX5, BCL6-associated germinal-center programs, and other lineage-maintenance genes, thereby contributing to incomplete terminal differentiation and differentiation arrest in lymphoma.

In DLBCL, PRDM1β overexpression has been associated with aggressive disease, and altered promoter methylation contributes to PRDM1α/PRDM1β imbalance (21). However, the upstream signals that selectively activate the PRDM1β promoter remain unclear. Although NF-κB, IRF4, inflammatory signaling, and oncogenic pathways may regulate PRDM1 expression more broadly in lymphoid malignancies, direct evidence that these pathways specifically drive PRDM1β promoter activation remains limited.

### 2.2 PRDM1 in B-cell differentiation

**Figure 2:**
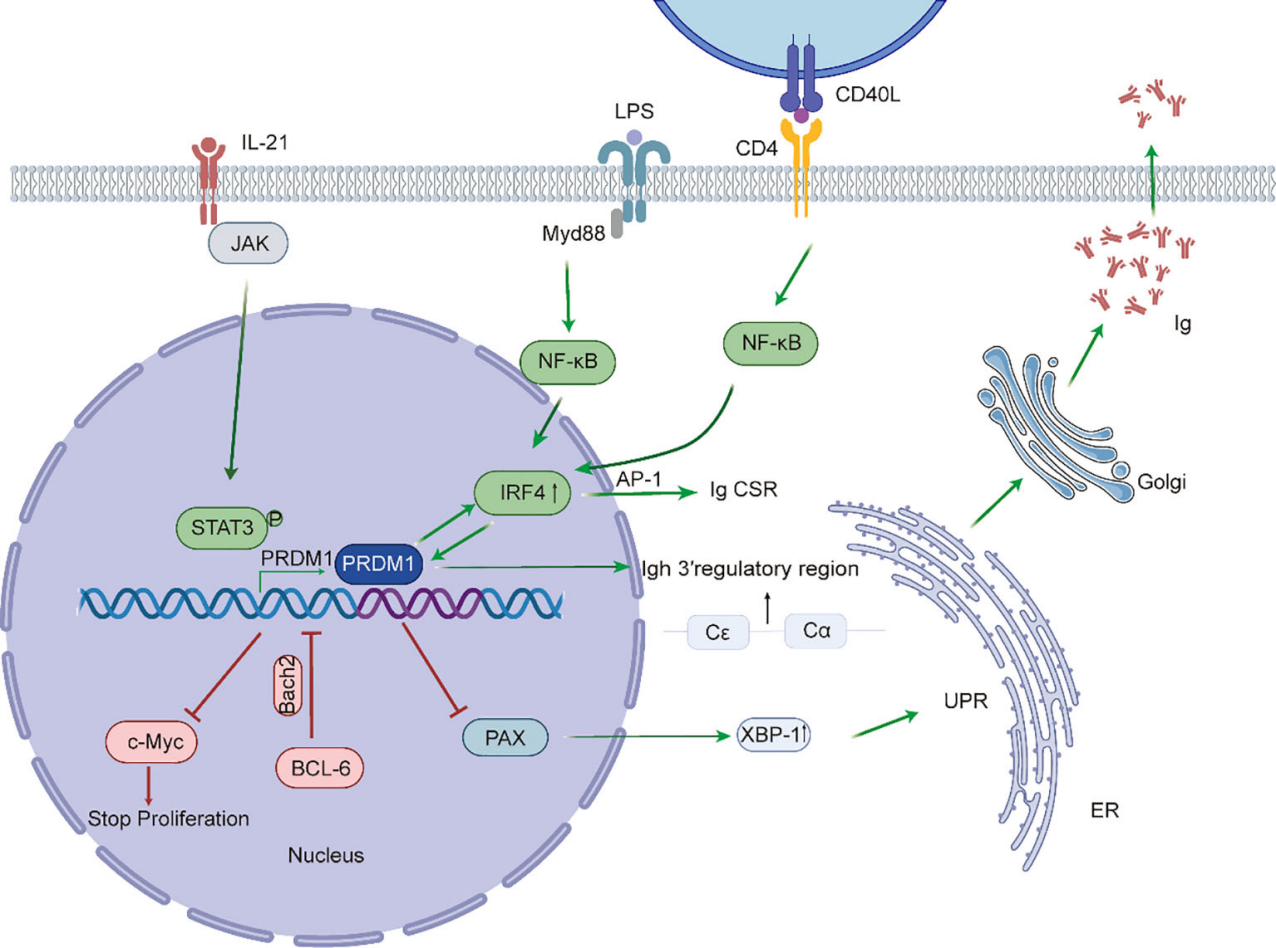
Regulatory network of PRDM1 in B-cell differentiation. PRDM1 integrates IL-21/JAK/STAT3, TLR/MyD88/NF-κB, and CD40L/CD40 signaling to regulate terminal B-cell differentiation. IRF4 promotes PRDM1 expression and cooperates with AP-1 during class-switch recombination. PRDM1 suppresses MYC, BCL6, Bach2, and PAX5 to inhibit proliferation and germinal center programs while promoting plasma cell differentiation. Repression of PAX5 facilitates XBP1 activation, which induces the unfolded protein response, ER/Golgi expansion, and immunoglobulin secretion. PRDM1 also contributes to immunoglobulin production by regulating the Igh 3′ regulatory region (3′RR), thereby supporting antibody synthesis.

Nutt et al. provided a transcriptional framework in which plasma-cell differentiation is driven by a coordinated switch from a B-cell/germinal-center program to an antibody-secreting-cell program. In this network, *PAX5, BCL6, BACH2* and *IRF8* maintain B-cell identity and prevent premature terminal differentiation, whereas *IRF4*, *PRDM1* and *XBP1* cooperate to establish plasma-cell fate and secretory function (22). *PRDM1* is a crucial regulator of B-cell differentiation into plasma cells. After B cells receive differentiation signals, such as antigen stimulation or regulatory cytokines, *PRDM1* expression is upregulated, initiating the plasma cell differentiation program (23). To further investigate the regulatory mechanisms of PRDM1, Shapiro-Shelef et al. using PRDM1^flox/flox^CD19^Cre/+^ mice revealed that although the number and subtypes of mature B cells were normal, the number of antibody-secreting cells and serum immunoglobulin levels were significantly reduced (24). Further research showed that PRDM1 inhibits *Pax5*, indirectly inducing *XBP-1* expression. XBP-1 plays a role in the unfolded protein response (UPR) within cells, enhancing endoplasmic reticulum function and promoting antibody synthesis and secretion. Although XBP1-deficient B cells can still differentiate into plasma cells, these cells exhibit impaired endoplasmic reticulum expansion and fail to secrete large amounts of immunoglobulins, indicating that XBP1 is essential for terminal secretory function rather than plasma-cell fate commitment (25). Therefore, PRDM1 may also have other targets involved in B-cell differentiation regulation (24, 26, 27). Subsequent studies found that c-Myc, a transcription factor usually associated with cell proliferation, is suppressed during terminal B-cell differentiation, indicating its close association with B-cell differentiation. To explore the underlying mechanisms, Lin et al. induced PRDM1 expression using IL-2 and IL-5, and discovered that PRDM1 binds to the plasmacytoma repressor factor (PRF) site on the c-Myc promoter, inhibiting its transcription and thereby halting B-cell proliferation and initiating differentiation (28). PRDM1’s regulation of B-cell differentiation is complex. Sean et al. constructed the STAT3-estrogen receptor fusion protein (STAT3-ER) system and used 4-hydroxytamoxifen (4-HT) to specifically activate STAT3.Compared to IL-21-activated JAK/STAT3 B cells, results showed that STAT3 upregulated PRDM1 expression, thereby promoting B-cell differentiation and enhancing antibody secretion (29).In addition to the JAK/STAT3 pathway, CD40L can also upregulate IRF4 through NF-κB activation. IRF4, together with AP-1, participates in B-cell immunoglobulin class switching, enhancing immunoglobulin secretion. CD40L also inhibits BCL-6 and, together with IL-21, maximizes PRDM1 upregulation, driving plasma cell differentiation (30). Minnich et al. provided direct evidence that PRDM1 activates IRF4 transcription during plasmablast differentiation. RNA-seq analysis showed that Irf4 expression was reduced in Prdm1-deficient pre-plasmablasts, while Bio-ChIP-seq demonstrated direct PRDM1 binding at regulatory regions of the IRF4 locus. In addition to transcription factor activation, PRDM1 directly contributed to immunoglobulin heavy-chain locus (Igh) activation. Genome-wide binding and chromatin analyses showed that PRDM1 associated with the 3′ regulatory region (3′RR) enhancers of the Igh locus, as well as regulatory elements of the Igk locus, thereby promoting large-scale immunoglobulin gene transcription during plasmablast maturation (18). Studies have also found that BCL-6 is widely expressed in germinal center B cells and acts as a negative regulator of plasma cell differentiation by maintaining B-cell proliferation and preventing premature differentiation. BCL-6 and Bach2 cooperate to suppress PRDM1 expression, while PRDM1 also suppresses BCL-6 expression. This relationship helps maintain the balance of B-cell differentiation (31). Muto et al. further clarified the regulatory role of BACH2 in this network. In Bach2-deficient splenic B cells, *PRDM1* was induced earlier and more strongly, leading to premature IgM plasma-cell differentiation; importantly, genetic loss of PRDM1 in Bach2-deficient B cells restored class-switch recombination. These findings demonstrate that BACH2 delays PRDM1 induction, thereby preventing premature plasma-cell differentiation and allowing class-switch recombination to occur before terminal differentiation (32). Toll-like receptors (TLRs) are also involved in B-cell differentiation. TLRs recognize bacterial lipopolysaccharide (LPS) and activate MyD88-dependent signaling, activating NF-κB and enhancing IRF4, which in turn promotes B-cell differentiation (33). Therefore, PRDM1 primarily regulates B-cell differentiation by controlling genes such as *MYC*, *Pax5*, *XBP-1*, and *BCL-6*. *PRDM1* expression is also regulated by various signaling pathways, particularly the upregulation of IRF4, STAT3, CD40L, and TLRs signaling pathways (**Figure 2**).

### 2.3 PRDM1 in T-cell function regulation

PRDM1 is also crucial for regulating T-cell function. Bankoti et al. using PRDM1-YFP mice and flow cytometry analysis revealed that PRDM1 is highly expressed in both regulatory T cells (Treg) and effector T cells (Teff) (34). In a Foxp3-Cre–mediated Prdm1 conditional knockout mouse model, Prdm1 was specifically ablated in Treg cells, leading to a significant expansion of Treg cell populations and increased Ki-67 expression, accompanied by mild intestinal inflammation. These findings suggest that loss of Prdm1 impairs the suppressive function of Treg cells, thereby compromising immune homeostasis. In CD4-Cre Prdm1 conditional knockout mice, Prdm1 deletion led to a significant increase in Teff cell numbers and pro-inflammatory cytokine production, resulting in chronic intestinal inflammation. Therefore, PRDM1 is critical for maintaining immune balance (34).Similar studies using PRDM1-YFP mice found that following infection with lymphocytic choriomeningitis virus (LCMV), PRDM1 expression significantly increased in CD8^+^ T cells, promoting effector T cell cytotoxic function. In PRDM1-knockout mice, effector T cells were more likely to differentiate into memory T cells, suggesting that PRDM1 regulates the balance between terminal differentiation and memory T cell formation (35).

Follicular helper T cells (TFH), a subset of CD4^+^ T cells that assist germinal center (GC) B cells, showed reduced differentiation upon IL-2 activation of STAT5. However, knocking down PRDM1 restored TFH differentiation, indicating that the inhibitory effect of STAT5 on TFH differentiation depends on PRDM1 (36). PRDM1 further regulates T-cell fate decisions by controlling the balance between follicular helper, effector, memory, and exhausted states. In CD4^+^ T cells, Crotty summarized that TFH cells require BCL6 and express *CXCR5*, *PD-1*, and *IL-21*, whereas *PRDM1* is low or absent in TFH cells but enriched in non-TFH effector CD4^+^ T cells. This indicates that *PRDM1* antagonizes the BCL6-dependent TFH program and promotes the differentiation of activated CD4^+^ T cells toward non-TFH effector fates (37). In CD8^+^ T cells, Kallies et al. showed that *PRDM1*is induced during antiviral responses and is required for terminal cytotoxic effector differentiation; loss of Blimp-1 weakens effector-cell formation and favors memory-associated features. This suggests that PRDM1 promotes terminal effector differentiation while limiting memory potential (38). However, Shin et al. demonstrated that during chronic viral infection, sustained high *PRDM1*expression promotes CD8^+^ T-cell exhaustion by increasing inhibitory receptor expression and suppressing normal memory differentiation, whereas intermediate *PRDM1* expression helps maintain residual effector function (39). Together, these studies show that *PRDM1* is not simply a positive or negative regulator of T-cell activation, but rather a dose- and context-dependent transcriptional regulator that determines whether activated T cells develop into TFH cells, terminal effector cells, memory-like cells, or exhausted T cells. This also explains why PRDM1 can support protective effector responses during acute infection but contribute to functional impairment under chronic antigen stimulation.

Recent studies have highlighted the complex, isoform-specific regulation of PRDM family members during T lymphocyte activation. PRDM1 exists as two major isoforms, PR+ PRDM1α, containing the N-terminal PR domain and exhibiting strong transcriptional repression, and PR− PRDM1β, lacking most of the PR domain with markedly reduced repressive activity. Similarly, PRDM2 (RIZ) produces PR+ RIZ1 and PR− RIZ2 isoforms through alternative promoter usage. In primary human CD4^+^ T cells, activation via TCR stimulation triggers a temporally coordinated induction of these transcripts, early activation favors PR− isoforms (RIZ2 and PRDM1β), supporting rapid proliferation and initial activation, whereas later stages show a progressive shift toward PR+ isoforms (RIZ1 and PRDM1α), promoting terminal differentiation, regulatory T cell stability, and effector function. Overexpression experiments further suggest that PRDM2 isoforms may influence PRDM1 isoform balance, with RIZ1 favoring PRDM1α and RIZ2 favoring PRDM1β, implying a potential upstream regulatory role of PRDM2 in modulating PRDM1 transcription (40).PRDM1 also possesses transcriptional activating functions. Guo et al. demonstrated that PRDM1 directly activates *FOXP3* by binding to its upstream enhancer and simultaneously promotes *FOXP3* expression indirectly through upregulation of *KLF2*, thereby enhancing Treg differentiation (41).

### 2.4 PRDM1 in non-immune tissues

PRDM1 not only plays a vital role in the immune system but also in various non-immune tissues. Research has shown that PRDM1 is crucial for uterine repair after pregnancy termination. *PRDM1* expression significantly increases in uterine tissues post-pregnancy, upregulating extracellular matrix-degrading enzymes such as Mmp10 and Mmp13, and regulating the degradation and remodeling of uterine stroma, promoting tissue breakdown and absorption (42). Additionally, PRDM1 plays a critical role in embryonic development. Studies have shown that the absence of PRDM1 results in a significant reduction in the number and distribution of primordial germ cells, leading to abnormal development of the second and third branchial arches in mice (43). Thus, PRDM1 has important roles in the development and differentiation of various tissues (44).

## 3 PRDM1 and cancer

PRDM1, a critical transcriptional repressor, plays multifaceted regulatory roles across diverse cancer types. In a context-dependent manner, PRDM1 may function either as a tumor suppressor or as an oncogenic driver. Such functional heterogeneity is largely influenced by cellular context, the activation status of signaling pathways, and the tumor microenvironment. In the following sections, the roles of PRDM1 and its underlying molecular mechanisms are systematically summarized across distinct cancer types.

### 3.1 Hematological tumors

#### 3.1.1 Diffuse large B-cell lymphoma

The PRDM1 gene is located on the 6q21-q22 region, which is a common deletion area in B-cell lymphoma. Numerous studies have confirmed the inactivation of PRDM1 and its role as a tumor suppressor gene in ALCL, with low PRDM1 expression being associated with poor prognosis in DLBCL patients (45–47). Pasqualucci et al. found that in 34 cases of DLBCL, 8 cases of ABC-DLBCL had inactivation of PRDM1 due to structural changes such as gene mutations and splice site mutations, leading to the loss of PRDM1 protein. In contrast, no PRDM1 deletions were found in GCB subtype and unclassified DLBCL (48). Further research through retrospective cohort studies of 199 DLBCL patients found that PRDM1 gene deletions were significantly associated with shorter survival in non-GCB-DLBCL (49). The loss of PRDM1 may promote the development of ABC-DLBCL by preventing B-cell differentiation into plasma cells, and further exploration of its mechanisms is of significant clinical importance.

**Figure 3:**
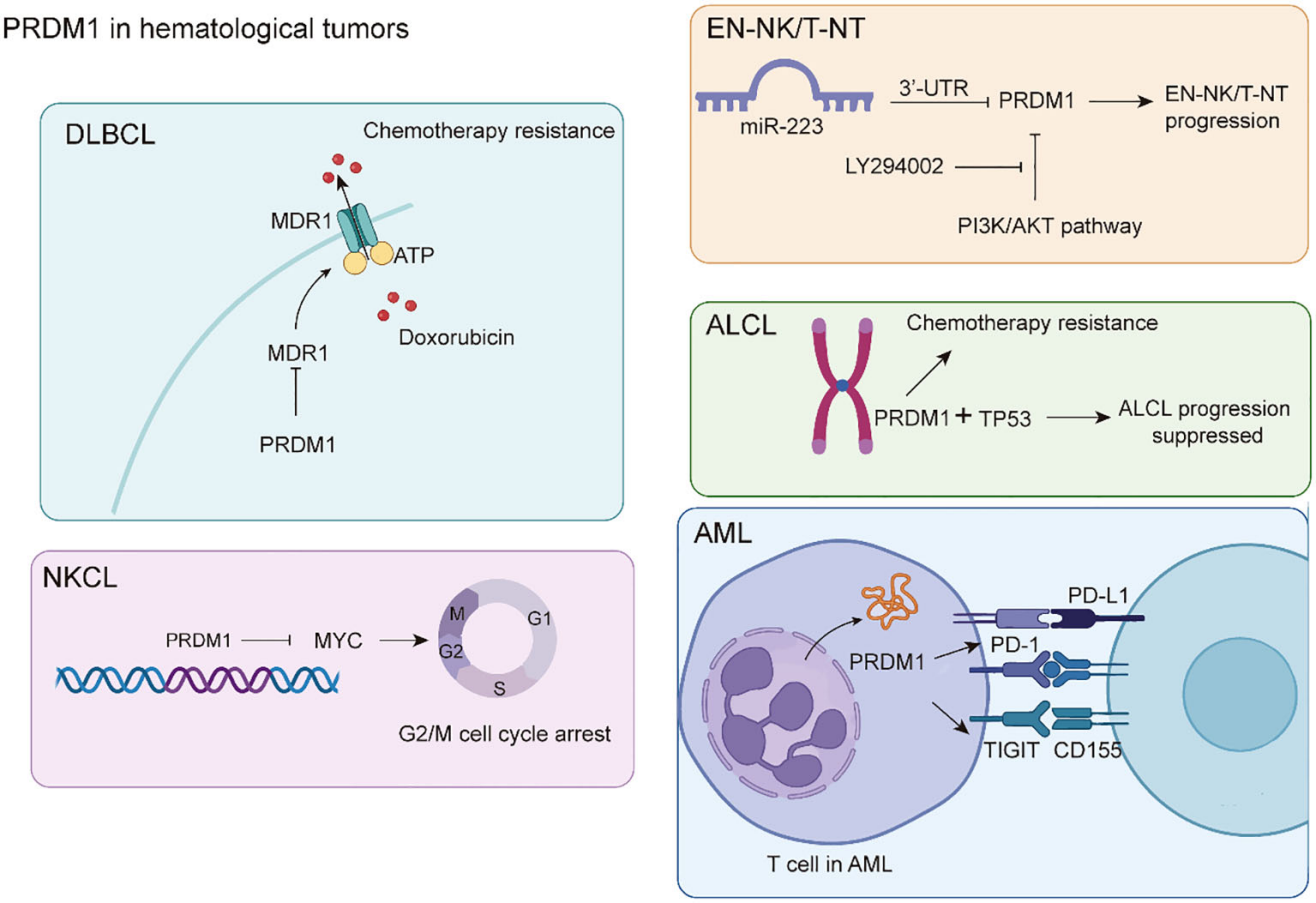
Context-dependent roles of PRDM1 in hematological malignancies. This schematic summarizes the major regulatory roles of PRDM1 in representative hematological malignancies. In DLBCL, PRDM1 downregulation is associated with MDR1-mediated chemotherapy resistance. In EN-NK/T-NT, miR-223-mediated suppression of PRDM1 and dysregulation of the PI3K/AKT pathway contribute to tumor progression. In ALCL, loss of PRDM1 is associated with lymphoma progression, whereas PRDM1 restoration suppresses tumor growth and promotes apoptosis. In NK-cell lymphoma (NKCL), PRDM1 represses MYC and induces G2/M cell-cycle arrest. In AML, PRDM1 promotes PD-1 and TIGIT expression in T cells, contributing to T-cell exhaustion and imp aired anti-leukemia immunity. .

In DLBCL cell lines, knockdown of PRDM1 leads to upregulation of c-Myc expression, thereby promoting B-cell proliferation, which may play an important role in the development of DLBCL (50). Studies have also found that PRDM1 isoforms exist in multiple myeloma cells and in non-GCB-DLBCL cells, where an imbalance between PRDM1α and PRDM1β expression occurs. PRDM1α is silenced by methylation, while PRDM1β is activated due to low methylation, and its transcriptional repression activity is weaker. This imbalance is closely related to tumor development (21). Studies suggest that PRDM1 not only participates in the development of DLBCL but also contributes to chemotherapy resistance in non-GCB-DLBCL cells. For example, in doxorubicin-resistant OCI-Ly3 cells, MDR1 expression was significantly upregulated. MDR1 is an ATP-dependent drug efflux pump that reduces the accumulation of chemotherapy drugs in cells. Further experiments revealed that NF-κB downregulates PRDM1 expression, leading to upregulation of MDR1 and promoting chemotherapy resistance. Upregulation of PRDM1 expression could help overcome chemotherapy resistance in non-GCB-DLBCL (51). Investigating the regulatory mechanisms of PRDM1, particularly its isoform expression imbalance, gene deletions, and their relationship with chemotherapy resistance, will be critical for improving DLBCL treatment strategies (**Table 1**; **Figure 3**).

**Table 1:** Summary of PRDM1 functions in cancer.

Cancer	Status	Regulators	Effects	Evidence	Ref.
DLBCL	Downregulated	MYC, MDR1	B-cell differentiation, proliferation	Cell experiments, Genetic analyses	(51)
EN-NK/T-NT	Downregulated	miR-223, PI3K/AKT	Proliferation, Cell-cycle	IHC, Cell experiments	(52)
NKCL	Downregulated	MYC	G2/M arrest	Cell experiments, Epigenetic analyses	(53)
AML	Upregulated	PD-1, TIGIT	T-cell exhaustion	T-cell functional assays	(54)
HCC	Upregulated	USP22/SPI1/PD-L1	Immune evasion	Cell experiments, Mouse models	(55)
CRC	Downregulated	P53, miR-223-3p, GSK3β/Wnt/β-catenin	Proliferation, Migration	Clinical cohorts, RNA-seq, Cell experiments	(56, 57)
BRCA	Upregulated	p130Cas/ErbB2/MAPK, TGF-β/c-Raf/Erk/AP-1, miR-23b, FAK, MP-5/Snail	EMT	Cell experiment, Mouse models	(58, 59)
Thyroid cancer	Upregulated	USP15, SELENBP1, CCL2/CCL5, CXCL10/CXCL11	Proliferation, Migration	Cell experiments	(60)
Lung cancer	Downregulated	LOX-PP-Ras/c-Raf/AP-1 pathway	Invasion, Apoptosis	Cell experiment, Mouse metastasis models	(61)
Melanoma	Downregulated	SOX10	Precursor proliferation	Cell experiments	(62)
Glioma	Downregulated	miR-30a-5p, MLL4, miR-27b-3p, Dkk1-Wnt/β-catenin, IL33	Proliferation, Metastasis	Cell experiments, Epigenetic mechanistic assays	(63, 64)
Bladder cancer	Downregulated	KPNA2/CBX8/BCOR, c-FOS	Proliferation, Migration	Cell experiments	(65)
Cervical Intraepithelial Neoplasia	Upregulated	–	–	IHC cohort, Clinical follow-up	(66)
PDAC	Upregulated	HIF1α, GLUT1, PGF	Metastasis	Mouse model Hypoxia, experiments	(67)

#### 3.1.2 Anaplastic large T-cell lymphoma

Anaplastic large T-cell lymphoma (ALCL) is a mature T-cell lymphoma in which PRDM1 has been shown to play a suppressive role in several immune-related tumors. Genomic analysis has revealed common deletions of the 6q21 region in ALCL patients, including the PRDM1. PRDM1 expression significantly inhibits ALCL cell growth and enhances apoptosis, demonstrating its tumor suppressive role in ALCL. A subsequent study found that the combined deletion of PRDM1 and TP53 significantly impacts patient survival, further proving PRDM1’s tumor suppressive role in ALCL and suggesting its potential as a clinical prognostic marker (68).

PRDM1’s role in ALCL chemotherapy resistance has also been a point of focus. In contrast to DLBCL, PRDM1 expression is typically lower in T-cell lymphoma. PRDM1-positive T-cell lymphoma cells exhibit stronger resistance to chemotherapy drugs such as doxorubicin. By inhibiting NF-κB activation with proteasome inhibitors, PRDM1 expression is reduced, leading to increased sensitivity to chemotherapy drugs. This suggests that PRDM1 plays a crucial role in chemotherapy resistance in T-cell lymphomas (69). The precise role of PRDM1 in ALCL remains unclear, and further research into its mechanisms, particularly its relationship with chemotherapy resistance, will provide new targeted strategies for ALCL treatment (**Table 1**; **Figure 3**).

#### 3.1.3 Extranodal NK/T-cell lymphoma

Extranodal NK/T-cell lymphoma (EN-NK/T-NT) is an aggressive malignancy, predominantly affecting the nasal cavity and other peripheral organs. Its pathological features include vascular destructive growth and necrotizing granulomatous degeneration, and it has a high mortality rate, particularly in East Asia and Central and South America (70). In a study of 43 cases of EN-NK/T-NT, fluorescence *in situ* hybridization (FISH) revealed that 55.81% of cases had heterozygous deletions of 6q21 and/or PRDM1. Among these, 16 cases had 6q21 deletions, 19 cases had PRDM1 deletions, and 11 cases exhibited concurrent deletions of both 6q21 and PRDM1. In cases with deletions of both 6q21 and PRDM1, only 16.67% showed PRDM1-positive staining, whereas in cases without deletions, 47.38% showed PRDM1-positive staining. This suggests that downregulation of PRDM1 expression is associated with gene deletion. Furthermore, PRDM1 expression levels were positively correlated with overall survival (OS) and progression-free survival (PFS), indicating that PRDM1 may serve as a prognostic marker for EN-NK/T-NT (71).

Further research revealed that miR-223 was highly expressed in EN-NK/T-NT patient samples, whereas PRDM1 was downregulated. miR-223 was found to bind to the 3’ untranslated region (UTR) of PRDM1, inhibiting its translation and possibly leading to the downregulation of PRDM1 in EN-NK/T-NT. As a tumor suppressor gene, PRDM1 inactivation is closely related to the development of EN-NK/T-NT (72). Additionally, the PI3K/AKT pathway is abnormally activated in this disease. Inhibition of the PI3K/AKT pathway using LY294002 increased PRDM1 and PTEN expression, significantly inhibiting EN-NK/T-NT cell proliferation and inducing cell cycle arrest (52). Future research should further investigate the mechanisms of PRDM1 inactivation and explore how targeting miR-223 or the PI3K/AKT pathway could improve EN-NK/T-NT treatment outcomes (**Table 1**; **Figure 3**).

#### 3.1.4 NK cell lymphoma

NK cell lymphoma (NKCL) is a highly aggressive non-Hodgkin lymphoma, and PRDM1 loss is associated with a highly methylated promoter region in NKCL patients. This suggests that *PRDM1* expression is epigenetically regulated. After overexpression of *PRDM1*, it was found to bind to the promoters of MYC, inhibiting their transcription and causing a G2/M cell cycle arrest and significant apoptosis in NKCL cells (73). Furthermore, PRDM1 is confirmed to function as a tumor suppressor gene in NK-cell malignancies. In studies where *PRDM1* was knocked out in NK cells, *MYC* expression increased, cell proliferation was enhanced, and apoptosis was reduced (53). Subsequent genome-wide comparative analysis using oligo-array and gene expression profiling revealed frequent deletions of the 6q21 region, which includes PRDM1, in NK cell malignancies, further confirming the role of PRDM1 as a tumor suppressor gene in NK cell malignancies (74, 75). This confirms the tumor suppressive role of PRDM1 in NK-cell malignancies and provides a new direction for molecular targeted therapy (**Table 1**; **Figure 3**).

#### 3.1.5 Leukemia

PRDM1 is highly expressed in acute myeloid leukemia (AML) compared to healthy controls, with more peripheral leukemia progenitor cells present. PRDM1 directly binds to the promoters of *PD-1* and *TIGIT*, regulating their transcription and promoting the expression of PD-1 and TIGIT, which leads to a significant decrease in cytokine production and cytotoxic function. Knockdown of PRDM1 restores T cell function in AML patients, indicating that PRDM1 plays a key role in immune escape in AML (54). Additionally, PRDM1 negatively regulates IL-2 expression. In CD4^+^ T cells with PRDM1 knockdown, IL-2 receptor expression, and the phosphorylation levels of its downstream signaling molecules (STAT5, ERK, and AKT) were significantly elevated. In ATL patients, the downregulation of PRDM1 expression in CD4^+^ T cells leads to increased IL-2 production and upregulation of proliferation-associated genes, potentially contributing to T-cell sustained proliferation (76) (**Table 1**; **Figure 3**).

### 3.2 Solid tumors

#### 3.2.1 Hepatocellular carcinoma

**Figure 4:**
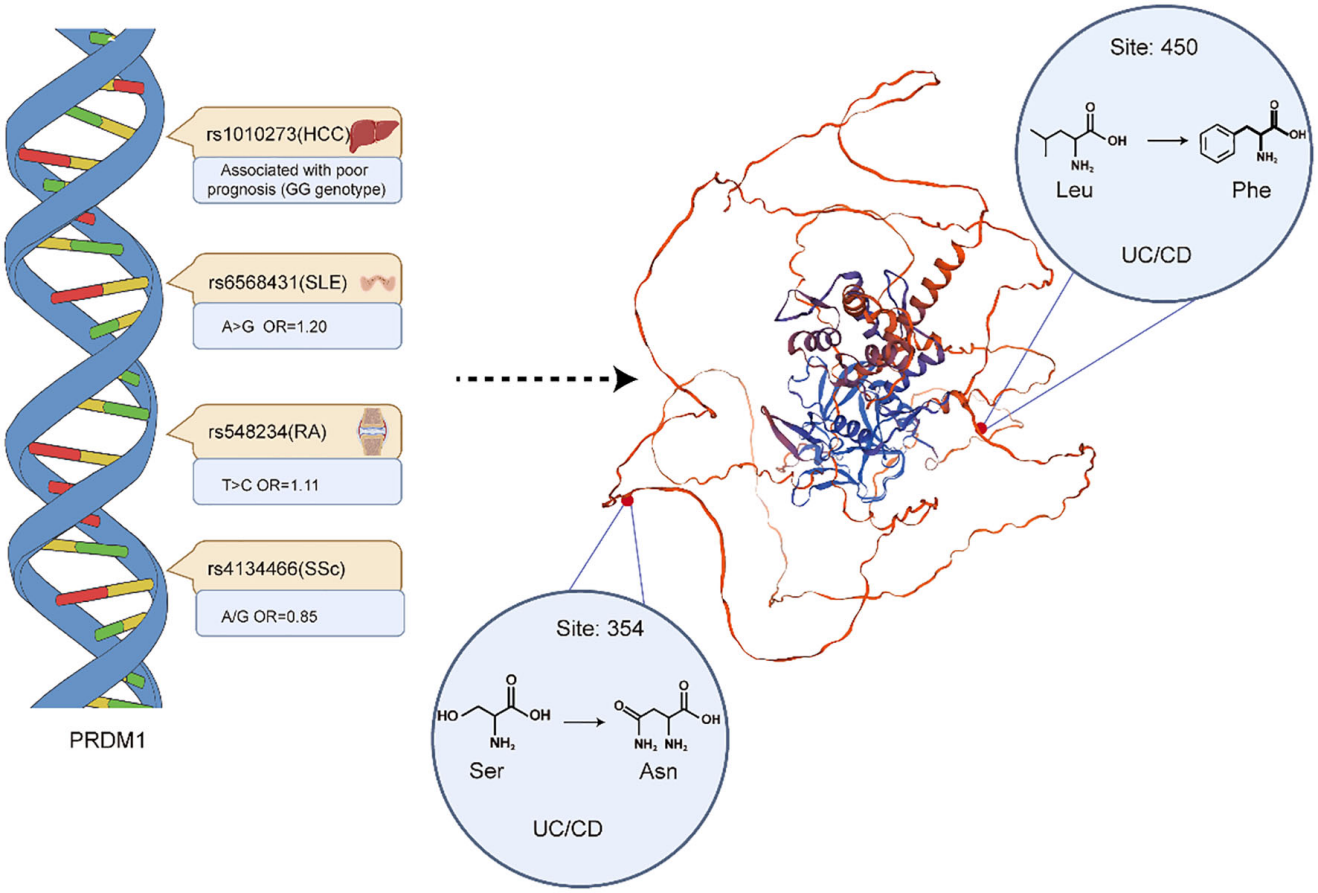
Disease-associated PRDM1 polymorphisms and structural alterations. This schematic summarizes representative PRDM1 SNPs associated with disease susceptibility and prognosis. The left panel shows selected PRDM1 variants linked to HCC, SLE, RA, and SSc, while the right panel illustrates predicted PRDM1 protein structural changes caused by representative amino acid substitutions at sites 354 and 450.

**Figure 5:**
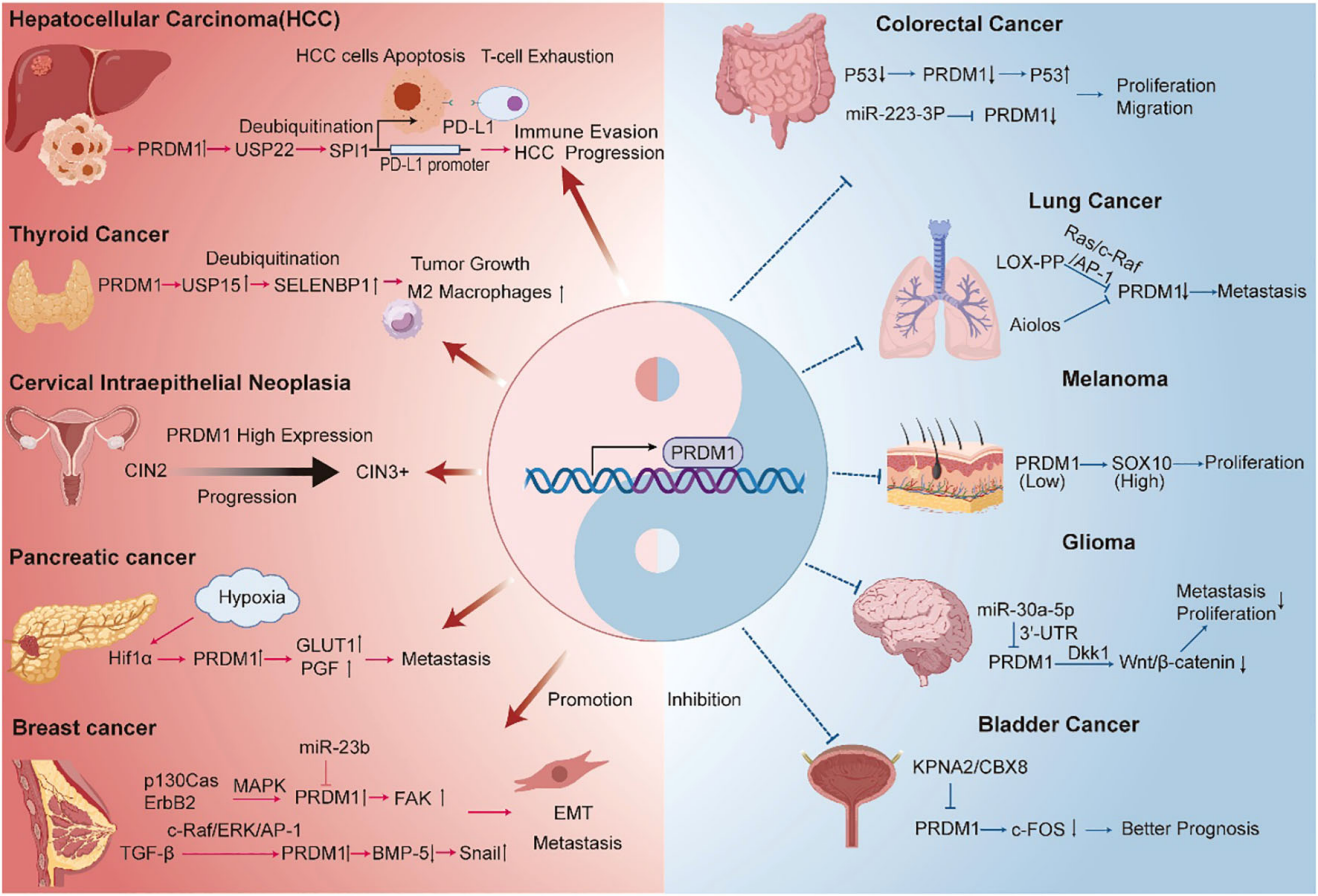
Dual and context-dependent roles of PRDM1 in solid tumors. This schematic summarizes the context-dependent regulatory roles of PRDM1 across representative solid tumors. In hepatocellular carcinoma (HCC), thyroid cancer, cervical intraepithelial neoplasia (CIN), pancreatic cancer, and breast cancer, PRDM1 is associated with tumor progression through regulation of immune evasion, hypoxia adaptation, metastasis, and epithelial-mesenchymal transition (EMT). In contrast, in colorectal cancer, lung cancer, melanoma, glioma, and bladder cancer, PRDM1 predominantly exerts tumor-suppressive effects by regulating cell proliferation, stemness, migration, metastasis, and oncogenic signaling pathways, including MYC, SOX10, Wnt/β-catenin, IL-33, and c-FOS. Collectively, PRDM1 exhibits dual and disease-specific functions in solid tumors, acting as either a tumor promoter or suppressor depending on the cellular and microenvironmental context.

Hepatitis B virus (HBV) infection is a severe global public health issue, and chronic HBV infection may eventually lead to hepatocellular carcinoma (HCC) (77). A study by Li et al. enrolled 403 chronic HBV patients, including 171 with chronic hepatitis, 119 with cirrhosis, and 113 with HCC. They found that PRDM1 levels were significantly higher in HCC patients compared to those with other liver diseases, suggesting that PRDM1 could be an effective molecular marker to distinguish HCC from other liver conditions (78). Subsequently, it was found that the PRDM1 single nucleotide polymorphism (SNP) variant rs1010273 GG genotype is significantly associated with poor prognosis in patients with HBV-related hepatocellular carcinoma (**Figure 4**), although its underlying mechanism still requires further investigation (79). Recent studies have shown that PRDM1 transcriptionally upregulates USP22, which deubiquitinates SPI1, thereby directly enhancing the activity of the PD-L1 promoter and leading to cell death. This suggests that PRDM1 acts as a tumor suppressor. However, *in vivo*, PRDM1-induced upregulation of PD-L1 expression can inhibit and induce exhaustion in CD8^+^ T cells, which may counteract the direct tumor-suppressive effects of PRDM1. Therefore, overexpression of PRDM1 in combination with PD-1 antibody therapy could potentially treat HCC. However, only a small fraction of HCC patients responds to PD-1 therapy (55). Overall, PRDM1 not only has potential as a molecular biomarker for early diagnosis of liver cancer but may also serve as a novel therapeutic target for its treatment (**Table 1**; **Figure 5**).

#### 3.2.2 Colorectal cancer

Colorectal cancer (CRC) has a high incidence and mortality rate globally, and the function of PRDM1 in CRC remains unclear. Slattery et al., based on RNA-seq analysis of 217 paired CRC tissues and adjacent normal mucosa, identified PRDM1 as one of the significantly downregulated tumor suppressor-associated genes in CRC (80). Consistently, low PRDM1 expression was closely associated with unfavorable survival outcomes in colon cancer patients, further supporting its potential tumor-suppressive role (81). Early work by Yan et al. revealed a negative feedback loop between PRDM1 and p53. Specifically, p53 directly binds to the *PRDM1* promoter and induces its transcription, whereas PRDM1, in turn, binds to the p53 promoter and represses p53 transcriptional activity. *PRDM1* knockdown resulted in marked upregulation of p53 and its downstream target genes, leading to cell cycle arrest and apoptosis in HCT116 colon cancer cells, suggesting that PRDM1 may sustain tumor cell survival by restricting excessive p53 activation (56). Subsequent studies further demonstrated that the function of PRDM1 is highly dependent on the TP53 genetic background. Weige et al., using TP53 Pro72Arg allele-specific models, showed that PRDM1β is a TP53 Arg72-induced transcript that promotes stem cell differentiation through epigenetic repression mechanisms, including H3K9me3-associated chromatin remodeling (82). In parallel, Liu et al. confirmed in colon cancer cells and organoid models that PRDM1β itself is a p53-responsive transcript. Both PRDM1α and PRDM1β were shown to suppress *MYC* and stem cell-related gene expression, while forced *PRDM1* expression markedly inhibited colon tumor organoid formation and clonal expansion (81). These findings suggest that under p53-activated conditions, PRDM1 preferentially exerts tumor-suppressive effects by limiting stemness maintenance and proliferative capacity.

However, the role of PRDM1 in CRC is not exclusively tumor suppressive. Kim et al. demonstrated that under ribosomal stress, activation of the PKR/eIF2α stress signaling axis induces *PRDM1* upregulation. In this context, PRDM1 no longer functions primarily as a suppressor of proliferation, but instead enhances ERK and GSK3β/Wnt/β-catenin signaling, thereby promoting tumor cell survival and chemoresistance (57). Beyond its tumor cell-intrinsic functions, PRDM1 also plays an important role in the CRC immune microenvironment. PRDM1 serves as a key transcription factor for effector regulatory T cells. Ward-Hartstonge et al. reported that PRDM1^+^ Treg infiltration improved the predictive value of immunoscore for disease-free survival (DFS) in CRC patients (83). Furthermore, high-dimensional mass cytometry analysis identified PRDM1^+^ Tregs as one of the most prominently enriched T-cell subsets within CRC tumor tissues, characterized by elevated expression of immunosuppressive and activation-associated molecules, suggesting a role in local immune regulation and tumor-associated immune tolerance (84). In addition, PRDM1 is subject to upstream post-transcriptional regulation. miR-223-3p, which is significantly upregulated in CRC, directly targets and suppresses PRDM1, thereby promoting tumor cell proliferation, migration, invasion, and epithelial–mesenchymal transition (EMT) (85). Conversely, the natural compound GD-1 induces *PRDM1* expression through the PKD1/MEK signaling pathway, leading to *MYC* downregulation and *p21* upregulation, ultimately suppressing cell cycle progression and proliferation in SW620 cells (86).

Collectively, PRDM1 appears to exert a highly context-dependent role in colorectal cancer rather than functioning as a conventional unidirectional tumor suppressor. Under basal or TP53-activated conditions, PRDM1 is generally associated with tumor-suppressive effects by inhibiting MYC-driven proliferation, limiting stemness maintenance, and restraining clonal expansion, highlighting its role as an important downstream regulator of TP53 signaling in CRC. However, under ribosomal stress, PRDM1 can shift toward promoting adaptive tumor survival by enhancing ERK and GSK3β/Wnt/β-catenin signaling, thereby facilitating chemoresistance and stress tolerance. In addition to its tumor-intrinsic effects, PRDM1 also contributes to CRC progression through modulation of the immune microenvironment, particularly by supporting immunosuppressive PRDM1^+^ Treg populations that may promote immune tolerance and tumor escape. Therefore, the biological role of PRDM1 in CRC is likely determined by TP53 status, cellular stress conditions, and microenvironmental context. This functional duality suggests that PRDM1 may serve not only as a potential biomarker for CRC progression and therapeutic response, but also as a context-dependent target requiring precise stratification in future translational studies (**Table 1**; **Figure 5**).

#### 3.2.3 Breast cancer

PRDM1 is closely associated with breast cancer metastasis and invasion. A study using p130Cas/ErbB2- overexpressing MCF10A.B2 breast epithelial cells cultured in 3D showed significant upregulation of PRDM1 in invasive acini. p130Cas/ErbB2 upregulated PRDM1 through the MAPK signaling pathway. PRDM1, in turn, upregulates focal adhesion kinase (FAK), promoting the degradation of the extracellular matrix. In mouse models, knockdown of PRDM1 significantly slowed tumor growth and reduced lung metastasis, suggesting that PRDM1 promotes breast cancer metastasis. *In vivo*, miR-23b inhibited PRDM1 expression, significantly reducing cell migration (59). PRDM1 plays a critical role in breast cancer metastasis, and studies have also shown its involvement in the TGF-β-induced epithelial-to-mesenchymal transition (EMT) process. Research revealed that TGF-β activates PRDM1 through the c-Raf/Erk/AP-1 signaling pathway, and PRDM1, by repressing BMP-5, induces the expression of Snail, a key regulator of EMT, thus promoting breast cancer cell migration (58). These findings not only enrich the understanding of the mechanisms by which PRDM1 contributes to breast cancer metastasis but also suggest new potential therapeutic targets for breast cancer (**Table 1**; **Figure 5**).

#### 3.2.4 Thyroid cancer

The thyroid is the organ with the highest selenium content in the body. Studies have shown that selenium is closely associated with the occurrence and development of thyroid cancer (TC). Selenium-binding protein 1 (SELENBP1) is highly expressed in TC tissues and cells, and its high expression correlates with clinical features and poor prognosis in TC patients. Knockdown of SELENBP1 inhibits the proliferation, migration, and invasion of TC cells, induces iron accumulation, and promotes ferroptosis, thus suppressing tumor growth. In addition, SELENBP1 regulates the immune microenvironment. Knockdown of *SELENBP1* reduces the expression of *CCL2*, *CCL5*, and M2 macrophage markers, while enhancing the expression of *CXCL10* and *CXCL11*. Furthermore, PRDM1 upregulates SELENBP1 expression via USP15. Therefore, PRDM1 promotes thyroid cancer cell growth, metastasis, and immune evasion, and the PRDM1/USP15/SELENBP1 regulatory mechanism offers potential directions for future targeted therapeutic strategies (60) (**Table 1**; **Figure 5**).

#### 3.2.5 Lung cancer

Lung cancer has the highest incidence and mortality rate globally. PRDM1 is typically considered a tumor suppressor. Zhu et al. found that low expression of PRDM1 in lung cancer was associated with poor prognosis. In the A549 lung cancer cell line, knockdown of PRDM1 significantly increased cell invasion and anti-apoptotic capacity. In *in vivo* experiments, injection of PRDM1-knockdown A549 cells significantly increased the incidence of lung metastasis, and mouse survival was shortened. Subsequent experiments revealed that Aiolos downregulated PRDM1 expression by inhibiting the promoter region of PRDM1 (87). Yu et al., however, found that lysyl oxidase propeptide (LOX-PP) inhibits the Ras/c-Raf/AP-1 pathway, thus suppressing *PRDM1* expression and weakening the metastasis and invasion of lung cancer cells (61). PRDM1 may exhibit different roles in different tumor microenvironments or signaling pathways, and its molecular mechanism in lung cancer development still needs further investigation (**Table 1**; **Figure 5**).

#### 3.2.6 Melanoma

Melanoma, an aggressive tumor originating from melanocytes, shows significantly lower expression of PRDM1 compared to normal melanocytes, and low expression of PRDM1 is associated with poor prognosis. PRDM1 inhibits Sox10 expression, and PRDM1 deletion leads to the overexpression of Sox10, which may cause excessive proliferation of melanocyte precursor cells, thereby promoting tumor development (62). By regulating PRDM1 or Sox10 expression or their related pathways, new therapeutic targets for melanoma treatment may be identified (**Table 1**; **Figure 5**).

#### 3.2.7 Glioma

Glioma is a common malignant tumor of the central nervous system with poor prognosis. PRDM1 is expressed at low levels in gliomas and its expression is negatively correlated with glioma grade. Wang et al. discovered that PRDM1 inhibits glioma proliferation and metastasis by suppressing the Wnt/β-catenin signaling pathway via Dkk1. MiR-30a-5p can bind to the 3’ untranslated region of PRDM1, inhibiting its expression and promoting glioma development. These findings provide new molecular targets for glioma therapy (63). Zhao et al. demonstrated that mixed linked leukemia 4(MLL4) enhances PRDM1 transcription by increasing H3K4me1 enrichment at the PRDM1 enhancer region. Subsequently, PRDM1 exerts transcriptional repressive activity by recruiting EHMT2 (G9a) to promote H3K9me3 deposition, thereby suppressing IL33 expression. Given that IL-33 has been reported to enhance glioma cell invasion and maintain glioma stem cell (GSC) stemness, these findings suggest that PRDM1 may exert a tumor-suppressive role in glioma cells by restricting IL33-driven malignant progression. In addition, M2-polarized tumor-associated macrophages (M2-TAMs) can suppress MLL4 expression through exosomal delivery of miR-27b-3p, which subsequently downregulates *PRDM1* and contributes to the maintenance of GSC stemness (64). On the other hand, Liu et al. reported that under a hypoxic microenvironment, increased *HIF1A* expression was accompanied by marked upregulation of *PRDM1*, together with elevated expression of *PD-L1* and other T-cell exhaustion-associated molecules, suggesting that PRDM1 may also participate in T-cell exhaustion and immune evasion, particularly within immune-related contexts (88). Notably, hypoxia itself is a well-established driver of M2-TAM polarization. Therefore, the hypoxia–HIF1A–M2-TAM axis may cooperatively reshape PRDM1-associated transcriptional networks, thereby contributing to the establishment of an immunosuppressive glioma microenvironment and promoting tumor progression. Future studies are needed to further determine whether HIF1A directly regulates *PRDM1* transcription, and whether PRDM1 itself is involved in the development of immunotherapy resistance in glioma (**Table 1**; **Figure 5**).

#### 3.2.8 Pancreatic cancer

Pancreatic ductal adenocarcinoma (PDAC) is a highly invasive malignant tumor, and its metastasis mechanism is unclear. Studies suggest that PRDM1 is an important driver of pancreatic cancer metastasis. In a mouse model of pancreatic cancer, metastatic cancer cells were labeled, and *PRDM1* expression was found to be high in cells with high metastatic potential, often located in hypoxic regions. It was later confirmed that under hypoxic conditions, HIF1α induces PRDM1 expression, which then regulates genes related to metastasis, such as GLUT1 and PGF, promoting PDAC cell metastasis. Furthermore, knockdown of PRDM1 significantly reduced PDAC cell metastatic potential, but overexpression of PRDM1 did not significantly alter metastatic ability, indicating that other regulatory factors are involved. This finding offers new insights for targeted therapy strategies aimed at the hypoxic tumor microenvironment in pancreatic cancer (67) (**Table 1**; **Figure 5**).

#### 3.2.9 Bladder cancer

PRDM1 acts as a tumor suppressor gene in bladder cancer. The interaction between KPNA2 and CBX8 recruits BCOR, thereby inhibiting *PRDM1* expression. PRDM1 inhibits the expression of downstream oncogene *c-FOS*, reducing bladder cancer cell proliferation, promoting apoptosis, and decreasing migration and invasion, thereby slowing bladder cancer progression. PRDM1 not only serves as a potential biomarker for bladder cancer diagnosis and prognosis but also suggests that KPNA2 and CBX8 can be potential therapeutic targets. Targeting the CBX8-PRDM1-c-FOS regulatory pathway holds significant basic research and clinical value (65) (**Table 1**; **Figure 5**).

#### 3.2.10 Cervical intraepithelial neoplasia

Cervical cancer is a common malignant tumor in women. In a study of 69 CIN2 cases, immunohistochemical staining of tissues showed significantly higher PRDM1 expression compared to normal cervical tissues. The number of CD4^+^ T cells in the lower layer of the lesion was significantly reduced. A subsequent follow-up of these 69 patients revealed that those with high *PRDM1* expression were more likely to progress to CIN3+, with higher sensitivity (85%) and specificity (79%). Therefore, high *PRDM1* expression is considered an independent prognostic factor for predicting the progression of CIN2 to CIN3+ (66) (**Table 1**; **Figure 5**).

## 4 Inflammatory/immune-related diseases

Aberrant expression or dysfunction of PRDM1 in inflammatory and autoimmune diseases can lead to the breakdown of immune tolerance and sustained activation of chronic inflammatory responses, thereby contributing to disease initiation and progression. For instance, in diseases such as systemic lupus erythematosus, rheumatoid arthritis, and inflammatory bowel disease, PRDM1 amplifies aberrant immune responses by regulating B-cell antibody production and T-cell inflammatory cytokine secretion. PRDM1 functions not only as a key regulator of immune homeostasis but also as an important contributor to the development of inflammatory and autoimmune disorders. A deeper understanding of its context-dependent roles across different immune cell types and microenvironments will be essential for elucidating the pathogenesis of immune-related diseases and for developing targeted therapeutic strategies.

### 4.1 PRDM1 in B-cell/plasma-cell-driven autoimmunity

#### 4.1.1 Systemic lupus erythematosus

**Figure 6:**
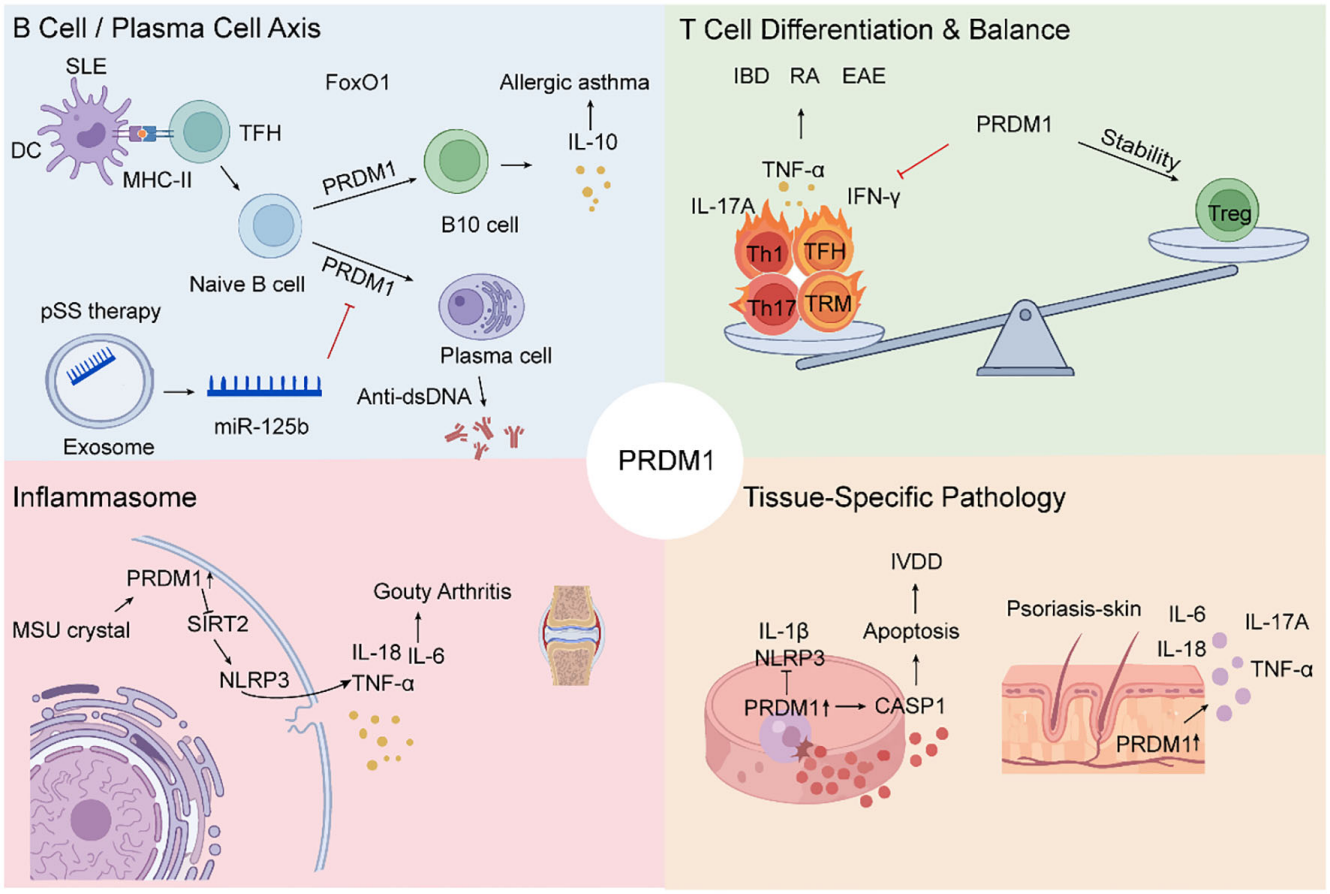
Major functions of PRDM1 in inflammatory and immune-related diseases. This schematic summarizes the major immunoregulatory functions of PRDM1 in inflammatory and immune-related diseases. In the B-cell/plasma cell axis, PRDM1 regulates plasma cell differentiation, antibody secretion, B10-cell formation, and IL-10 production, thereby influencing humoral immunity and immune tolerance. In T-cell differentiation and balance, PRDM1 maintains Treg stability and suppresses excessive Th1, Th17, TFH, and TRM-associated inflammatory responses, thereby modulating cytokines such as TNF-α, IL-17A, and IFN-γ. In the inflammasome pathway, PRDM1 participates in inflammatory regulation through SIRT2/NLRP3-associated signaling, influencing pro-inflammatory cytokine release. In tissue-specific pathological contexts, PRDM1 regulates CASP1 activation, apoptosis, and inflammatory cytokine production, contributing to disease progression in conditions such as psoriasis, gouty arthritis, and intervertebral disc degeneration (IVDD).

Systemic Lupus Erythematosus (SLE) is an autoimmune disease, and PRDM1 is closely related to its pathogenesis. Studies have found that early expression of PRDM1 in B cells promotes their differentiation into plasma cells, leading to an increased number of plasma cells. This mechanism is associated with the development of SLE and other autoimmune diseases (89). Preliminary studies have confirmed that PRDM1 is a susceptibility gene for SLE. The SNP site rs6568431 is significantly associated with SLE (*P* = 7.1 × 10^-10), with the A-to-G mutation increasing susceptibility to SLE and other autoimmune diseases (90) (**Figure 4**). In SLE patients and the MRL/lpr mouse model, PRDM1 expression was significantly elevated (*P* < 0.05), and its expression levels were positively correlated with disease activity and the secretion of autoantibodies (91). Sun et al. discovered that in SLE, the downregulation of PRDM1 expression in dendritic cells led to the abnormal upregulation of cathepsin S (CTSS), which enhanced MHC II-dependent antigen processing and presentation. The overactivation of CTSS altered the TCR repertoire of follicular helper T (TFH) cells, driving the production of autoantibodies and lupus-like phenotypes (92). Furthermore, Luo et al. confirmed that knocking down PRDM1 in the mouse model significantly reduced anti-dsDNA antibody production and alleviated immune damage and kidney lesions in SLE mice, while lowering urinary protein levels. These findings suggest that PRDM1 is a potential therapeutic target for SLE (93). PRDM1 plays a critical role in B-cell differentiation, antibody production, and the pathogenesis of SLE, and future studies can further explore its interactions with other cytokines in SLE to provide new treatment strategies (**Table 2**; **Figure 6**).

**Table 2:** Summary of PRDM1 functions in inflammatory and autoimmune diseases.

Disease type	Dominant cell type	Mechanism	Evidence	Ref.
Systemic Lupus Erythematosus	B cells/plasma cells	B-cell differentiation and autoantibody production	GWAS cohorts, Mouse model	(91)
Hashimoto’s Thyroiditis	B cells/plasma cells	B-cell/plasma-cell infiltration	Clinical samples	(89)
Allergic Asthma	B10 cells	B10-cell differentiation and worsening airway inflammation	Cell experiments, Mouse models	(94)
Primary Sjögren’s syndrome	B cells/plasma cells	Inhibits plasma-cell differentiation and reduces lymphocytic infiltration	Mouse model	(95)
Psoriasis	Th17-associated inflammatory axis	PRDM1 correlates with IL-17A, IL-6, TNF-α and IL-22	Patient lesion analysis2, Mouse model	(96)
Autoimmune Diabetes	Th1/Th17 cells	Reducing Th1/Th17-mediated β-cell destruction	NOD mouse model	(97)
Rheumatoid Arthritis	CD4^+^ T cells, Th17/Treg axis	Inhibits Th17 and promotes Treg differentiation	GWAS cohorts, Immune-cell studies	(98)
Autoimmune Encephalomyelitis	Treg cells, Teff, TFH cells	PRDM1 maintains Treg stability	EAE mouse model	(99)
Inflammatory Bowel Disease	CD4^+^ tissue-resident memory T cells	PRDM1 deletion promotes Th1/Th17 differentiation	Mouse model	(100)
Gouty Arthritis	Macrophages, Inflammasome pathway	PRDM1 inhibits SIRT2, increases α-tubulin acetylation and activates NLRP3 inflammasome	ChIP assay, Mouse model	(101)
Intervertebral Disc Degeneration	Nucleus pulposus cells	PRDM1 activates CASP1 transcription and promotes inflammatory cell death	RNA-seq, Gene-silencing assays	(102)
Atherosclerosis	T cells in plaques	PRDM1/RUNX3/IRF7 network disruption contributes to transition from stable to unstable plaques	Plaque expression analysis, Mouse model	(103)

#### 4.1.2 Hashimoto’s thyroiditis

Hashimoto’s thyroiditis (HT) is a common autoimmune disease, characterized by the degeneration of follicular epithelial cells and infiltration by B cells and plasma cells to form lymphoid follicles. PRDM1 is implicated in the pathogenesis of HT (89, 104). Studies have shown that after parvovirus B19 (PVB19) infection, its NS1 protein induces increased expression of *PRDM1*, contributing to HT development. This discovery provides a new direction for treatment strategies related to immune regulation in PVB19-associated HT (105) (**Table 2**).

#### 4.1.3 Allergic asthma

Allergic asthma is a chronic airway inflammatory disease caused by abnormal immune responses. B cells play an essential role in the immune response, especially IL-10-producing B cells (B10 cells), which have immunosuppressive effects and help alleviate airway inflammation. FoxO1, a negative regulator, inhibits *PRDM1* expression, suppressing B10 cell differentiation and exacerbating airway inflammation, thus promoting asthma development. Therefore, PRDM1 could become a new target for asthma treatment. Modulating PRDM1 function or its downstream pathways may help alleviate asthma symptoms (94) (**Table 2**; **Figure 6**).

#### 4.1.4 Primary Sjögren’s syndrome

Primary Sjögren’s syndrome (pSS) is a chronic autoimmune disease primarily affecting exocrine glands, leading to dry mouth and dry eyes. The disease is closely related to abnormal B-cell activation. Xing et al. used exosomes derived from labial gland mesenchymal stem cells (LGMSCs) to treat pSS in a mouse model, resulting in increased salivation and reduced lymphocytic infiltration. Dual luciferase reporter assays showed that miR-125b binds to the 3’ UTR region of *PRDM1*, inhibiting its expression, thereby inhibiting plasma cell differentiation and reducing inflammatory infiltration, offering potential therapeutic strategies for pSS (95) (**Table 2**; **Figure 6**).

### 4.2 T-cell-driven inflammatory disease

#### 4.2.1 Psoriasis

PRDM1 is significantly related to inflammatory skin diseases. In some eczema cases, *PRDM1* expression in skin keratinocytes is significantly reduced. In mouse models, *PRDM1* deletion leads to chronic skin inflammation and abnormal granulocyte proliferation, possibly offering new therapeutic strategies for immune-inflammatory skin diseases such as eczema and contact dermatitis (106). Psoriasis, a chronic immune-mediated skin disease, involves the IL-23/Th17 pathway. PRDM1 expression is upregulated in the skin lesions of psoriasis, especially in keratinocytes, and is closely associated with keratinocyte proliferation, abnormal keratinization, and disruption of skin barrier function. *PRDM1* expression is positively correlated with inflammatory cytokines such as IL-17A, IL-6, TNF-α, and IL-22, suggesting that PRDM1 may promote psoriasis inflammation by regulating the Th17 pathway. PRDM1, as a novel immune target for psoriasis, may provide new approaches for its treatment (96) (**Table 2**; **Figure 6**).

#### 4.2.2 Autoimmune diabetes

Autoimmune diabetes is related to the dysfunction of Th (helper T) cells, and PRDM1 plays a key role in regulating T-cell function. Studies have shown that in PRDM1 transgenic NOD mice, the incidence of islet inflammation and diabetes is lower than in controls. Additionally, the expression of *Tbx21*, *Ifng*, and differentiation-related genes in Th17 cells is suppressed, leading to impaired Th1 and Th17 differentiation and function. This decreases the destruction of β cells in the pancreas (97). PRDM1’s regulation of immune responses offers new insights into potential therapies for autoimmune diseases like diabetes (**Table 2**).

#### 4.2.3 Rheumatoid arthritis

PRDM1 is a crucial regulator in the immune pathology of rheumatoid arthritis (RA). GWAS has identified that the SNP site rs548234 in *PRDM1* is significantly associated with RA (*P* = 2.1 × 10^-8) (**Figure 4**). The T-to-C mutation at this site alters the function of the *PRDM1*, which may play a role in immune cell differentiation and immune regulation, thus influencing the risk of diseases like RA (107). PRDM1 promotes B-cell differentiation and antibody production in CD4^+^ T cells through regulating IL-21 secretion, affecting the interaction between T and B cells and promoting synovial inflammation (102, 108). Latest research suggests that PRDM1 may also be involved in immune regulation in RA through exosomes. PRDM1 regulates Th17 transcription factors, inhibiting Th17 cell differentiation and promoting Treg cell differentiation, thus restoring immune system balance and alleviating RA inflammation. This provides a new perspective for RA treatment (98) (**Table 2**).

#### 4.2.4 Inflammatory bowel disease

PRDM1 is closely related to the development of intestinal inflammation. Deletion of PRDM1 leads to excessive activation of dendritic cells, promoting Th1 and Th17 cell differentiation, which aggravates colonic inflammation (100). Whole-exome sequencing of Crohn’s disease (CD) and ulcerative colitis (UC) patients and healthy controls revealed that missense mutations at Ser354Asn and Leu450Phe in PRDM1 were significantly associated with CD and UC (*P* = 1.18 × 10^-3^, *P* = 5.88 × 10^-6^) (**Figure 4**). In Ser354Asn mutant CD patients, T-cell proliferation, IFN-γ secretion, and the expression of activation markers (CD69, CD44, CD25) were significantly increased, suggesting that the Ser354Asn mutation may lead to excessive T-cell activation, thereby aggravating CD inflammation (109). Further research revealed that compared to healthy controls, CD patients had significantly increased numbers of CD4^+^ tissue-resident memory T cells (TRM) in the gut, which secreted higher levels of cytokines, especially TNF-α and IL-17A. PRDM1 was highly expressed in TRM cells and promoted TNF-α and IL-17A production, driving immune responses in CD (110). These findings provide new insights into CD pathogenesis and suggest that PRDM1 may be a novel therapeutic target for CD (**Table 2**; **Figure 6**).

#### 4.2.5 Autoimmune encephalomyelitis

PRDM1’s functional regulation plays a crucial role in the onset and progression of experimental autoimmune encephalomyelitis (EAE). Under normal conditions, PRDM1 helps maintain the stability of Treg cells, ensuring immune system tolerance and preventing autoimmune responses. However, in the EAE mouse model, *PRDM1* deletion not only affects Treg cell stability but also promotes their differentiation into effector T cells, producing IL-17A and Granulocyte-macrophage colony-stimulating factor (GM-CSF), both of which are associated with neuroinflammation and autoimmunity. This also leads to excessive activation of TFH cells, promoting antibody production and worsening EAE symptoms (99). Thus, PRDM1 may become a potential therapeutic target for treating EAE and other autoimmune diseases (**Table 2**).

### 4.3 PRDM1 in tissue-intrinsic and stromal inflammatory responses

#### 4.3.1 Gouty arthritis

Gouty arthritis is induced by the deposition of monosodium urate (MSU) crystals, which activate macrophages and initiate inflammatory responses. MSU crystals activate the NLRP3 inflammasome, resulting in higher levels of NLRP3 inflammasome components and pro-inflammatory cytokines. This suggests that PRDM1 plays a key role in acute gouty arthritis. Research has shown that PRDM1 promotes the acetylation of α-tubulin by inhibiting SIRT2, which activates the NLRP3 inflammasome and exacerbates inflammation. ChIP experiments confirmed that PRDM1 binds to the promoter region of *SIRT2*, and overexpression of *PRDM1* in mice exacerbated gouty arthritis. This finding provides a potential therapeutic target for treating gouty arthritis (101) (**Table 2**; **Figure 6**).

#### 4.3.2 Intervertebral disc degeneration

*PRDM1* is highly expressed in degenerated nucleus pulposus cells of intervertebral discs compared to normal tissue. By inhibiting the expression of mitochondrial autophagy-related proteins (NLRP3, GSDMD-N, IL-1β), PRDM1 promotes nucleus pulposus cell death. RNA-seq analysis revealed that PRDM1 directly activates *CASP1* transcription, promoting necroptosis of nucleus pulposus cells and exacerbating intervertebral disc degeneration. Silencing *CASP1* eliminates PRDM1-mediated mitochondrial autophagy, indicating that PRDM1 exacerbates intervertebral disc degeneration (102) (**Table 2**; **Figure 6**).

#### 4.3.3 Atherosclerosis

Jin et al. used weighted gene co-expression network analysis (WGCNA) to identify T cells as key drivers in the progression of stable plaques to unstable plaques in atherosclerosis. The *PRDM1*, *RUNX3*, and *IRF7* gene network showed disrupted expression. Experimental data showed that compared to stable plaques, PRDM1 expression was downregulated in unstable plaques. In a mouse model of atherosclerosis, Prdm1^-/-^Ldlr^-/-^ mice developed larger, more advanced plaques. This suggests that PRDM1 plays a protective role in atherosclerosis (103) (**Table 2**).

#### 4.3.4 Systemic sclerosis

Systemic sclerosis (SSc) is an autoimmune disease characterized by widespread fibrosis, with high incidence and mortality rates (111). In 2017, Terao et al. conducted a transethnic meta-analysis of genome-wide association studies (GWAS) from Japan and Europe, analyzing 4,436 cases and 14,751 controls. The study identified *PRDM1* as one of the susceptibility genes for SSc. The SNP site rs4134466 in *PRDM1* was significantly associated with SSc, and the A/G genotype at this site has a protective effect on SSc (**Figure 4**). This finding expands our understanding of the genetic background of the disease and provides potential targets for further research (112).

## 5 Clinical applications

Adoptive immunotherapy is a strategy that uses a patient’s own immune cells for cancer treatment. In this therapy, gene editing is used to modify T cells to recognize and kill tumor cells. However, T cells often experience functional exhaustion or premature differentiation during the treatment process. Gene editing to knock out PRDM1 can effectively maintain the early memory phenotype of anti-tumor T cells, enhance their cytokine production capacity, and significantly improve T-cell persistence and anti-tumor efficacy *in vivo*. This discovery provides a new direction for improving adoptive immunotherapy, especially in CAR-T cell therapy. By targeting PRDM1 knockdown, it may help prevent premature differentiation and exhaustion of T cells, ultimately improving long-term therapeutic outcomes (113).

Subsequent studies in a multiple myeloma mouse model confirmed that *PRDM1* knockout CAR-T cells were significantly superior to the control group, significantly extending the mice’s survival, with tumor progression completely suppressed in some cases. These results provide a new treatment strategy for multiple myeloma patients (114). In addition to its potential in immunotherapy, PRDM1 is also closely related to the side effects of other cancer treatments. Hodgkin lymphoma patients undergoing radiation therapy face an increased risk of secondary malignant tumors (SMNs), particularly children treated with radiotherapy, who have a higher incidence of SMNs. A GWAS found that SNPs rs4946728 and rs1040411 in the 6q21 region are associated with SMNs. These mutations, through modulation of *PRDM1* expression and *MYC* inhibition, may affect radiation-induced tumor development (115).

## 6 Limitations of the current literature

Several limitations should be considered when interpreting current PRDM1-related studies. First, the available evidence is heterogeneous, including cell-line experiments, animal models, retrospective clinical cohorts, transcriptomic analyses, and SNP/GWAS studies, which provide different levels of mechanistic strength. Second, the evidence is uneven across diseases, PRDM1 has been relatively well studied in B-cell/plasma-cell differentiation and lymphoid malignancies, whereas many solid tumors and inflammatory diseases are supported mainly by correlative or disease-specific findings. Third, some clinical and genetic studies, particularly those involving HBV-related hepatocellular carcinoma, extranodal NK/T-cell lymphoma, and PRDM1 SNPs, are derived mainly from specific populations, especially East Asian cohorts. Therefore, these findings may not be fully generalizable and require validation in independent multi-ethnic cohorts. Fourth, PRDM1-related biomarkers, including SNPs, expression levels, promoter methylation, 6q21 deletion, and PRDM1α/PRDM1β imbalance, still require standardized assays, reproducible cut-off values, prospective validation, and demonstration of clinical utility. Finally, therapeutic translation remains challenging because PRDM1 is a transcription factor without an established druggable catalytic pocket. Indirect approaches targeting PRDM1-associated chromatin regulators or upstream pathways may be more feasible, but they require disease-specific and mechanism-based validation.

## 7 Future directions and prospects

Given its context-dependent roles in immune regulation, tumor progression, and epigenetic remodeling, PRDM1 has emerged as a promising candidate for precision biomarker development and therapeutic modulation. At the clinical level, PRDM1-related alterations—including single nucleotide polymorphisms (SNPs), 6q21 deletion, promoter hypermethylation, and PRDM1 isoform imbalance (PRDM1α/PRDM1β)—may serve as useful biomarkers for disease stratification, prognosis evaluation, and molecular classification in both hematologic malignancies and immune-related diseases. In addition, PRDM1-associated transcriptional programs, such as PD-1/PD-L1/TIGIT-related immune exhaustion signatures, may provide value for predicting tumor immune status and therapeutic responsiveness.

From a therapeutic perspective, context-dependent modulation of PRDM1 may be more feasible than direct universal targeting. In tumors where PRDM1 acts as a tumor suppressor, restoring PRDM1 activity through reversal of promoter hypermethylation or modulation of upstream inhibitory pathways may represent potential strategies. Conversely, in contexts where PRDM1 contributes to immune exhaustion, metastasis, or stress adaptation, targeting PRDM1-associated downstream pathways may be more clinically relevant. Since PRDM1 frequently functions through chromatin regulators such as G9a/EHMT2, HDACs, and transcriptional cofactor complexes, epigenetic intervention may offer an indirect but practical translational route. Furthermore, PRDM1 modulation in CAR-T-cell engineering has shown potential to maintain early memory phenotypes and improve anti-tumor persistence, highlighting its broader relevance in immunotherapy optimization. Overall, future studies should focus on precision stratification, pathway-specific intervention, epigenetic targeting, and immune engineering, thereby improving the translational value of PRDM1-based therapeutic strategies.

## Glossary

PRDM1: PR Domain zinc finger protein 1
PRDI-BF1: PRDI-binding factor I
Blimp-1: B lymphocyte-induced maturation protein-1
FOG1/FOG2: Friend of GATA-1/Friend of GATA-2, HDACs, Histone deacetylases
UPR: Unfolded protein response
XBP-1: X-box binding protein 1
PAX-5: Paired box protein 5
AP-1: Activator protein 1
IRF4: Interferon regulatory factor 4
MyD88: Myeloid differentiation primary response protein 88
NF-κB: Nuclear factor kappa B
BCL-6: B-cell lymphoma 6
Bach2: BTB and CNC homology 2
TLRs: Toll-like receptors
LPS: Lipopolysaccharide
TFH: Follicular helper T cells
FOXP3: Forkhead box P3
KLF2: Kruppel-like factor 2
Mmp10/Mmp13: Matrix metallopeptidase 10/Matrix metallopeptidase 13
DLBCL: Diffuse Large B-Cell Lymphoma
ABC-DLBCL: Activated B-cell-like diffuse large B-cell lymphoma
GCB-DLBCL: Germinal center B-cell-like diffuse large B-cell lymphoma
MDR1: Multidrug resistance protein 1
ALCL: Anaplastic large T-cell lymphoma
EN-NK/T-NT: Extranodal NK/T-cell lymphoma
PTEN: Phosphatase and tensin homolog
NKCL: NK cell lymphoma
AML: acute myeloid leukemia
TIGIT: T cell immunoreceptor with Ig and ITIM domains
ATL: Adult T-cell leukemia/lymphoma
HBV: Hepatitis B virus
HCC: hepatocellular carcinoma
SNP: single nucleotide polymorphism
USP22: Ubiquitin-specific peptidase 22
PD-1/PD-L1: Programmed cell death protein 1/Programmed death-ligand 1
CRC: Colorectal cancer
PKD1: Protein kinase D1
MEK: Mitogen-activated protein kinase
IGF: Insulin-like growth factor
pGSK3β: Phosphorylated glycogen synthase kinase 3 beta
PKR: Protein kinase R
MAPK: Mitogen-activated protein kinase
TGF-β: Transforming growth factor beta
BMP-5: Bone morphogenetic protein 5
EMT: Epithelial-to-mesenchymal transition
TC: Thyroid Cancer;SELENBP1, Selenium binding protein 1
USP15: Ubiquitin-specific peptidase 15
CCL2/CCL5L: C-C motif chemokine ligand 2/C-C motif chemokine ligand 5
CXCL10/CXCL11: C-X-C motif chemokine ligand 10/C-X-C motif
Dkk1: Dickkopf-related protein 1
PDAC: Pancreatic ductal adenocarcinoma
HIF1α: Hypoxia-inducible factor 1-alpha
GLUT1: Glucose transporter 1
PGF: Placental growth factor
KPNA2: Karyopherin subunit alpha 2
CBX8: Chromobox protein homolog 8
BCOR: BCL6 corepressor
SLE: Systemic Lupus Erythematosus
CTSS: Cathepsin S
HT: Hashimoto’s thyroiditis
NS1: Non-structural protein 1
MSU: Monosodium urate
NLRP3: NLR family pyrin domain containing 3
SIRT2: Sirtuin 2
SSc: Systemic sclerosis
Tbx21: T-box transcription factor 21
Ifng: Interferon gamma
pSS: Primary Sjögren’s syndrome
UTR: 3-prime untranslated region
RA: Rheumatoid Arthritis
GWAS: Genome-wide association study
GSDMD-N: Gasdermin D N-terminal fragment
CASP1: Caspase 1
RUNX3: Runt-related transcription factor 3
IRF7: Interferon regulatory factor 7
FoxO1: Forkhead box O1
UC/CD: Ulcerative colitis/Crohn’s disease
EAE: Experimental autoimmune encephalomyelitis
